# Aciculatin Inhibits Granulocyte Colony-Stimulating Factor Production by Human Interleukin 1β-Stimulated Fibroblast-Like Synoviocytes

**DOI:** 10.1371/journal.pone.0042389

**Published:** 2012-07-31

**Authors:** Kao-Shang Shih, Jyh-Horng Wang, Yi-Wen Wu, Che-Ming Teng, Chien-Chih Chen, Chia-Ron Yang

**Affiliations:** 1 Orthopedic Department, Shin Kong Wu Ho-Su Memorial Hospital, Taipei, Taiwan; 2 School of Medicine, Fu-Jen Catholic University, New Taipei City, Taiwan; 3 School of Medicine, Taipei Medical University, Taipei, Taiwan; 4 Department of Orthopedic Surgery, National Taiwan University Hospital, Taipei, Taiwan; 5 Institute of Pharmacology, College of Medicine, National Taiwan University, Taipei, Taiwan; 6 Department of Biotechnology, Hungkuang University, Taichung, Taiwan; 7 School of Pharmacy, College of Medicine, National Taiwan University, Taipei, Taiwan; College of Tropical Agriculture and Human Resources, University of Hawaii, United States of America

## Abstract

The expression of granulocyte colony-stimulating factor (G-CSF), the major regulator of neutrophil maturation, by human fibroblast-like synoviocytes (FLS) can be stimulated by the inflammatory cytokine interleukin-1β (IL-1β). G-CSF is known to contribute to the pathologic processes of destructive arthritis, but the induction mechanism remains unknown. The aims of this study were to identify the signaling pathways involved in IL-1β-stimulated G-CSF production and to determine whether this process was inhibited by aciculatin (8-((2*R*,4*S*,5*S*,6*R*)-tetrahydro-4,5-dihydroxy-6-methyl-2*H*-pyran-2-yl)-5-hydroxy-2-(4-hydroxyphenyl)-7-methoxy-4*H*-chromen-4-one), the major bioactive component of *Chrysopogon aciculatus*. IL-1β-induced cytokine expression was evaluated by measuring mRNA and protein levels by RT-PCR, ELISA, and Milliplex® assay. Whether aciculatin inhibited IL-1β-stimulated G-CSF expression, and if so, how, were evaluated using western blot assay, an electrophoretic mobility shift assay, and a reporter gene assay. Neutrophil differentiation was determined by Wright-Giemsa staining and flow cytometry. Aciculatin markedly inhibited G-CSF expression induced by IL-1β (10 ng/mL) in a concentration-dependent manner (1–10 µM). In clarifying the mechanisms involved, aciculatin was found to inhibit the IL-1β-induced activation of the IκB kinase (IKK)/IκB/nuclear factor-κB (NF-κB) and mitogen-activated protein kinase (MAPK) pathways by suppressing the DNA binding activity of the transcription factors NF-κB and activator protein (AP)-1. Furthermore, aciculatin significantly inhibited the G-CSF-mediated phosphorylation of Janus kinase-signal transducer and activator of transcription (JAK-STAT) and Akt and neutrophil differentiation from precursor cells. Our results show that aciculatin inhibits IL-1β-stimulated G-CSF expression and the subsequent neutrophil differentiation, suggesting that it might have therapeutic potential for inflammatory arthritis.

## Introduction

Osteoarthritis (OA) is a major arthritic disease. The current understanding of the molecular events that take place during joint destruction suggests that activated synoviocytes play important roles in the progression of OA [1]–[4]. Inflammatory mediators, e.g. interleukin-1β (IL-1β), produced by mononuclear cells that infiltrate into the synovial membrane and cause further inflammation, can be detected in OA synovial fluid, the synovial membrane, and subchondral bone and cartilage [1]. These mediators amplify and perpetuate the OA disease process by inducing catabolic inflammatory mediators, including cytokines [5], chemokines [6], and angiogenic factors [7], increasing mononuclear cell/macrophage infiltration [8], increasing levels of extracellular proteolytic enzymes involved in cartilage degradation, such as matrix metalloproteinases (MMPs) [9], and suppressing cartilage anabolism by decreasing glycosaminoglycan and collagen biosynthesis [10], [11]. Thus, inflammatory mediator-activated pathways appear attractive targets for the treatment of OA [1]–[4].

Granulocyte colony-stimulating factor (G-CSF) is the major regulator of granulocyte production [12], neutrophil maturation, and mobilization [13]; recombinant G-CSF has been used extensively to enable bone marrow transplantation and to treat chemotherapy-associated neutropenia [12], [13]. G-CSF is mainly produced by non-hematopoietic cells, including fibroblast-like synoviocytes (FLS), bone marrow stromal cells, endothelial cells, and macrophages, and its production is induced by inflammatory stimuli [12], [13]. Serum G-CSF levels are low under normal conditions, but increase markedly during an infection [14]. G-CSF binds to the G-CSF receptor (G-CSFR), and this results in the activation of the JAK-STAT (Janus kinase-signal transducer and activator of transcription (JAK-STAT) [15] and phosphatidylinositol 3-kinase (PI3K)-Akt [16] signaling pathways, which promote neutrophil survival, proliferation, adhesion, and myeloid differentiation. Recent studies suggest that G-CSF is an important modulator in inflammatory arthritis [13], [17]–[19], and G-CSF administration has been shown to exacerbate arthritis [20] by increasing the production and mobilization of myeloid lineage cells from the bone marrow [17] and inducing the expression of macrophage antigen-1 (Mac-1) and leukocyte function-associated antigen-1 (LFA-1) on neutrophils to promote leukocyte recruitment at sites of inflammation [17]–[19]. Serum and synovial fluid levels of G-CSF also correlate positively with the total histological score [21], [22]. In contrast, G-CSF-deficient mice are resistant to induction of inflammatory arthritis [17]. These results suggest that G-CSF levels could be a biomarker of inflammatory arthritis [22] and that G-CSF could be a therapeutic target [12], [13], [17]. Thus, the development of compounds with the ability to reduce G-CSF levels might be a rational strategy for the treatment of inflammatory arthritis.

Natural products are a valuable source of new therapeutic agents. Aciculatin (8-((2*R*,4*S*,5*S*,6*R*)-tetrahydro-4,5-dihydroxy-6-methyl-2*H*-pyran-2-yl)-5-hydroxy-2-(4-hydroxyphenyl)-7-methoxy-4*H*-chromen-4-one), isolated from whole *Chrysopogon aciculatus* plants, has been used as a traditional Chinese medicine to treat fever and the common cold for hundreds of years. Our previous study showed that aciculatin exerts a potent anti-inflammatory effect by inhibiting the expression of lipopolysaccharide-mediated inducible nitric oxide synthase and cyclooxygenase-2 [23]. However, the molecular details of the anti-arthritic effect of aciculatin, and whether it is effective against OA, are not known. The present study was performed to examine whether aciculatin inhibited G-CSF expression by IL-1β-stimulated FLS, and if so, to elucidate the underlying mechanism. Aciculatin was found to decrease IL-1β-induced G-CSF expression and G-CSF-associated neutrophil maturation, and these effects were correlated with its inhibitory effect on IL-1β-mediated nuclear factor (NF)-κB and mitogen-activated protein kinase (MAPK) activation. These findings suggest that aciculatin has potential as an anti-arthritic agent.

## Results

### Aciculatin Suppresses IL-1β-induced G-CSF Production by FLS

In all studies, unless stated otherwise, IL-1β was used at the concentration of 10 ng/mL and all inhibitor treatments consisted of a 30 min pre-incubation with FLS, followed by co-incubation of the FLS for the indicated time with the inhibitor and IL-1β.

**Figure 1:**
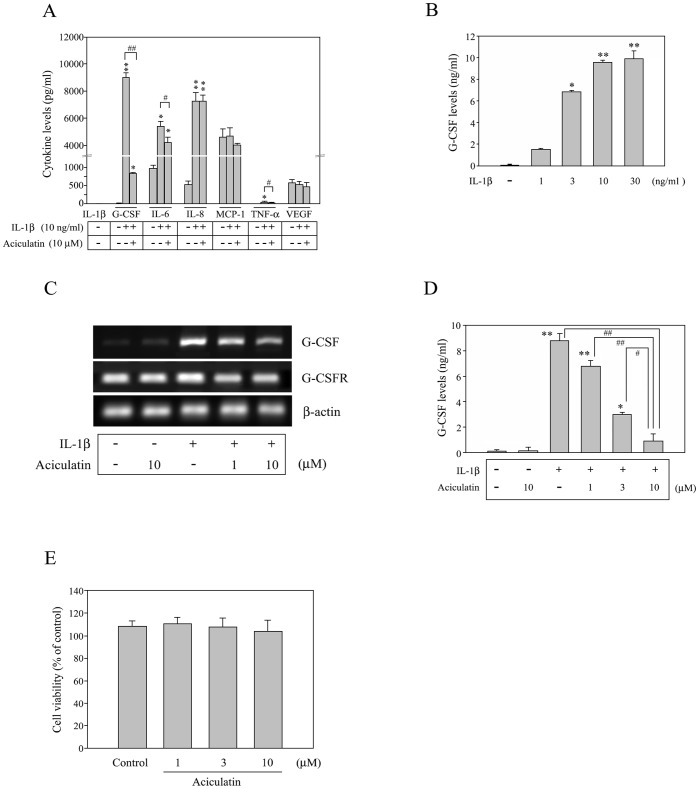
Aciculatin inhibits IL-1β-induced G-CSF production in a concentration-dependent manner. (A) 1×10^6^ fibroblast-like synoviocytes (FLS) were incubated with or without 10 µM aciculatin for 30 min and then for 24 h with or without 10 ng/mL of IL-1β in the continued presence of aciculatin; thereafter, cell culture supernatants were assayed for cytokine levels by using a Milliplex® assay. (B) FLS were incubated with 0–30 ng/mL of IL-1β for 24 h, and then the culture supernatants were assayed for G-CSF by using ELISA. (C) FLS were incubated with 0, 1, or 10 µM aciculatin for 30 min, and then for 5 h with 10 ng/mL of IL-1β in the continued presence of aciculatin. The G-CSF mRNA levels in the cells were measured using RT-PCR, and a control with only 10 µM aciculatin was included. (D) Cells were incubated for 30 min with 0–10 µM aciculatin and then for 24 h with 10 ng/mL of IL-1β in the continued presence of aciculatin, before the G-CSF in the culture supernatants were measured using ELISA. (E) The viability of the FLS was determined after 24 h treatment with 1–10 µM of aciculatin compared to the control group by using the MTT assay. Data are represented as mean ± SEM, with n = 3. *p<0.05 and **p<0.01 compared with the control group; #*p*<0.05 and ##*p*<0.01 for the comparisons of the groups indicated.

FLS treated with recombinant human IL-1β for 24 h were assessed for secretion of 7 cytokines by using a human cytokine Milliplex® assay. As shown in Figure 1A, endogenous IL-1β was undetectable in the culture medium before the addition of recombinant IL-1β. Following IL-1β stimulation, the levels of G-CSF, IL-6, IL-8, and tumor necrosis factor (TNF)-α were markedly increased in the cell supernatant while the levels of monocyte chemotactic protein (MCP)-1 and vascular endothelial growth factor (VEGF) were unchanged. The increase in G-CSF, IL-6, and TNF-α protein levels was significantly reduced by pretreatment and co-treatment with 10 µM aciculatin, with the inhibitory effect for G-CSF being the greatest. To further assess the inhibitory effect of aciculatin, FLS were incubated for 30 min with different concentrations (0–10 µM) of aciculatin, then with IL-1β for 24 h in the presence or absence of aciculatin. Thereafter, G-CSF levels in the culture medium were measured using ELISA. As shown in Figure 1B, little G-CSF was released in the absence of IL-1β. However, IL-1β treatment increased G-CSF expression in a concentration-dependent manner, with levels plateauing at 10 ng/mL and 30 ng/mL of IL-1β (9.56±0.32 ng/mL and 9.87±0.27 ng/mL of G-CSF, respectively). Interestingly, aciculatin pre-treatment inhibited the IL-1β-induced increase in the G-CSF mRNA levels in FLS (Figure 1C) and in G-CSF protein in the culture supernatant (Figure 1D) in a concentration-dependent manner. This inhibition was not due to downregulation of the G-CSF receptor (G-CSFR) or a decrease in cell viability since none of the treatments had any significant effect on G-CSFR mRNA levels (Figure 1C) or cell viability assessed using the MTT assay (Figure 1E).

### Aciculatin Inhibits NF-κB and MAPK Signaling in IL-1β-activated FLS

**Figure 2:**
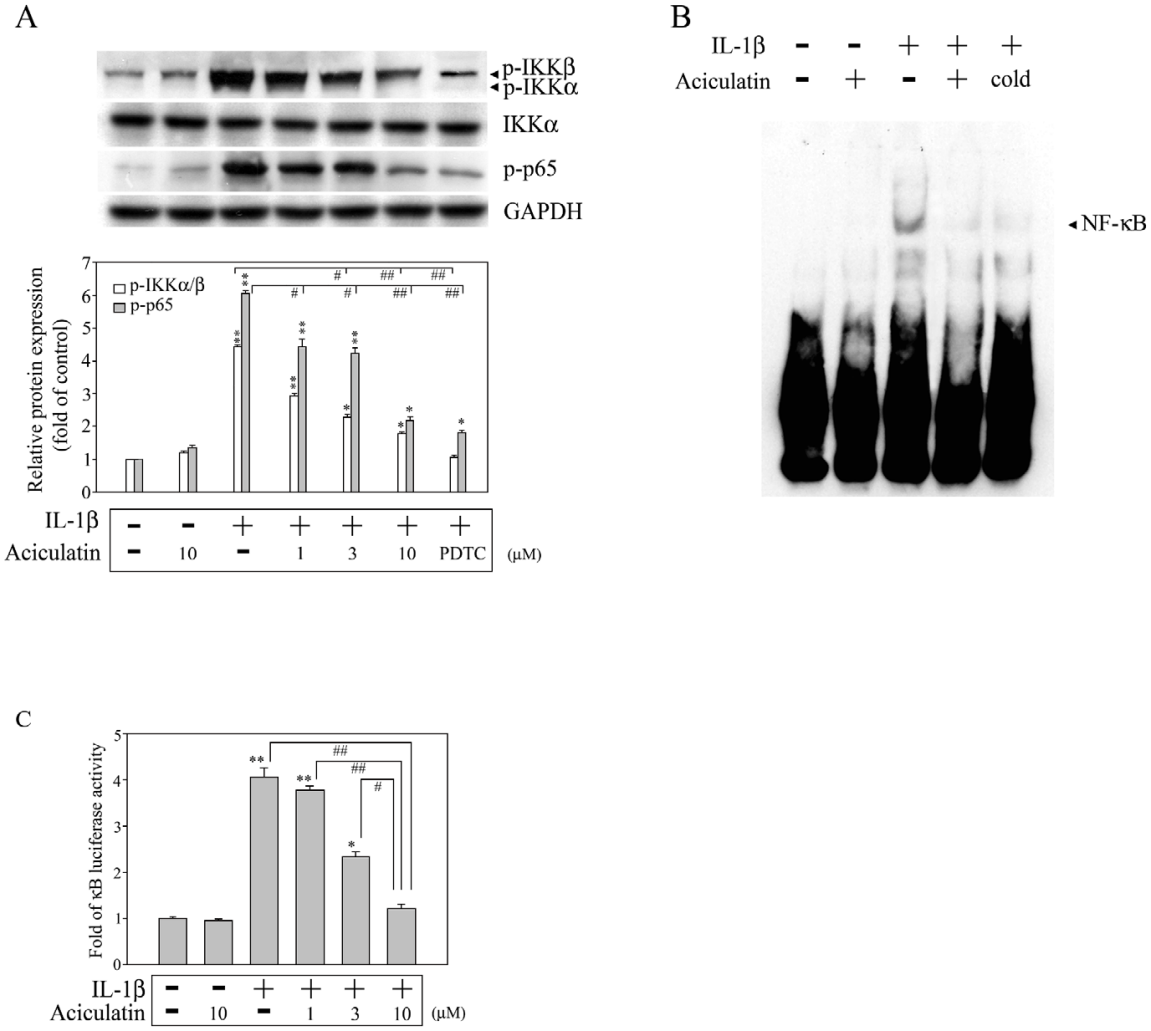
Aciculatin inhibits NF-κB activation in IL-1β-stimulated FLS. (A) FLS (1×10^6^ cells) were treated with 0–10 µM aciculatin or 20 µM PDTC for 30 min, and were then incubated for 30 min with 10 ng/mL of IL-1β in the continued presence of aciculatin or PDTC. The cells were then harvested, and whole cell extracts were subjected to western blot analysis for the indicated proteins. The bar graphs represent the ratio of phosphorylation of IKKα/β and of p65 proteins expression to the relative levels of GAPDH protein that were quantitated using a densitometer scanner and Image-Pro plus software. (B) FLS were incubated with 0 or 10 µM aciculatin for 30 min, and then for 1 h with 10 ng/mL of IL-1β in the continued presence of aciculatin. The DNA binding activity of the nuclear extracts was then examined in an electrophoretic mobility shift assay using a biotinylated NF-κB DNA probe; controls included 10 µM aciculatin alone and the use of the unlabeled probe (“cold”) to compete for the binding of the labeled probe. (C) Cells (1×10^5^ cells) were transiently transfected with 1 µg of pGL4.32[*luc2P/*NF-κB-RE*/*Hygro] for 24 h and incubated with 0–10 µM aciculatin for 30 min prior to stimulation for 6 h with 10 ng/mL of IL-1β in the continued presence of aciculatin, and then luciferase activity was measured. A control with only 10 µM aciculatin was used. In (A) and (C), the results are expressed as the mean ± SEM, with n = 3. *p<0.05 and **p<0.01 compared with the control group; #*p*<0.05 and ##*p*<0.01 for the comparisons of the groups indicated.

**Figure 3:**
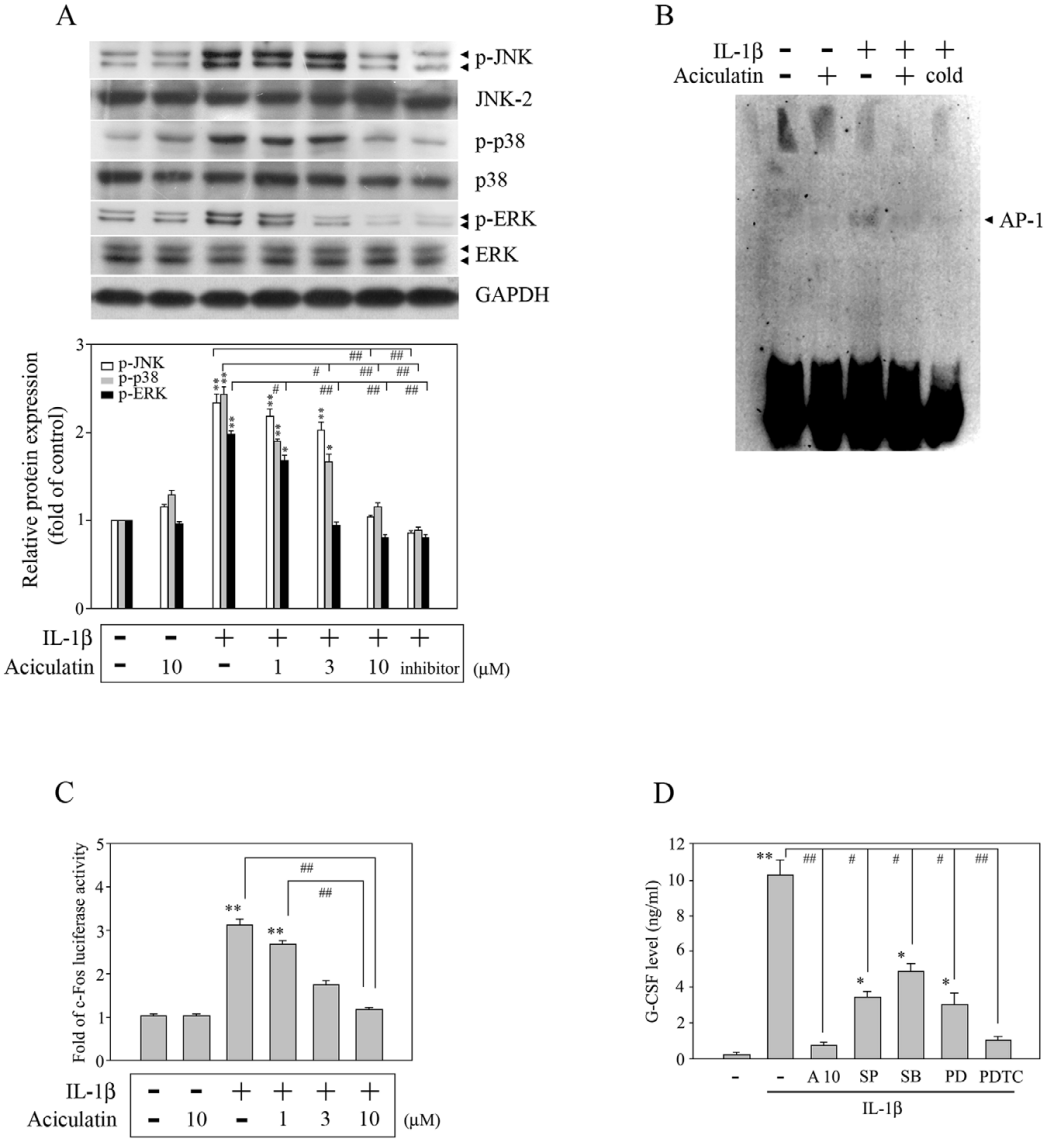
Aciculatin inhibits MAPK pathways. (A) Cells were incubated for 30 min with 0–10 µM aciculatin or 20 µM SP600125 (a JNK inhibitor), SB203580 (a p38 inhibitor), or PD98059 (an ERK inhibitor), and then for 30 min with 10 ng/mL of IL-1β in the continued presence of aciculatin or inhibitor; a control with only 10 µM aciculatin was used. The cells were then harvested, and whole cell extracts were prepared for western blot analysis of the indicated proteins. The extents of indicated proteins expression were quantitated using a densitometer with a scientific imaging system, and the relative levels were calculated as the ratios of indicated proteins to GAPDH protein levels. (B) The cells were incubated with 0 or 10 µM aciculatin for 30 min, and then for 1 h with 10 ng/mL IL-1β in the continued presence of aciculatin; a control with only 10 µM aciculatin was used. Nuclear extracts were then subjected to a DNA-binding reaction with biotinylated oligonucleotides specific for AP-1. The DNA binding activity of the AP-1 complex is indicated by an arrow. (C) Cells (1×10^5^ cells) were transiently transfected with 1 µg of p5xATF6-GL3 for 24 h, and then incubated with 0–10 µM aciculatin for 30 min. The cells were then incubated for 6 h with 10 ng/mL of IL-1β in the continued presence of aciculatin, following which luciferase activity was measured. (D) FLS were incubated for 30 min with a vehicle, 10 µM aciculatin, or different inhibitors as indicated (20 µM), and then for 24 h with 10 ng/mL of IL-1β in the continued presence of aciculatin or inhibitor, before the supernatants were assayed for G-CSF by ELISA. In (A), (C), and (D), the results are expressed as the mean ± SEM, with n = 3. *p<0.05 and **p<0.01 compared with the control group; #*p*<0.05 and ##*p*<0.01 for the comparisons of the groups indicated.

It is known that IL-1β can induce G-CSF expression in human synovial fibroblasts [24], [25], but the signaling cascade involved remains unknown. Previous studies have shown that the NF-κB and MAPK pathways play pivotal roles in regulating the expression of inflammatory mediators by IL-1β-stimulated FLS and are involved in the progression of inflammatory arthritis [26], [27]. To characterize the molecules involved in the inhibitory effect of aciculatin, we examined whether aciculatin had any effect on these signaling pathways. FLS were treated with 0–10 µM aciculatin for 30 min prior to stimulation with IL-1β in the continued presence of aciculatin for a further 30 min, then levels of phosphorylated or total IKKα/β and p65 were measured by western blotting. As shown in Figure 2A, IL-1β treatment resulted in significant phosphorylation of IKKα/β at serine 180/181 and of p65, and aciculatin pre-treatment markedly inhibited both effects in a concentration-dependent manner. An NF-κB inhibitor (pyrrolidine-dithiocarbamate; PDTC, 20 µM) treatment group was included as a positive control for the inhibition of IKKα/β and p65 phosphorylation. Furthermore, after 1 h of treatment with IL-1β, a significant increase in NF-κB DNA binding activity was seen in an electrophoretic mobility shift assay (EMSA), and this effect was inhibited by aciculatin (Figure 2B). The results of a promoter activity assay showed that aciculatin caused concentration-dependent inhibition of IL-1β-mediated NF-κB promoter activation (Figure 2C). In addition, a 30-min treatment with IL-1β also significantly increased the phosphorylation of JNK, p38, and ERK (Figure 3A), AP-1 DNA binding activity (Figure 3B), and c-fos promoter activation (Figure 3C); all of which were markedly inhibited by aciculatin (Figure 3A–C). Furthermore, when FLS were stimulated with IL-1β for 24 h, the induced G-CSF expression was inhibited by the addition of either aciculatin or inhibitors of JNK (SP600125), p38 (SB203580), ERK (PD98059), or NF-κB (PDTC) (Figure 3D). These results show that IL-1β induces G-CSF production via NF-κB- and MAPK-dependent pathways and suggest that aciculatin exerts its inhibitory effect by inhibiting these pathways.

### Aciculatin Suppresses G-CSF-mediated JAK/STAT Signaling and Differentiation of Neutrophils from Mouse Bone Marrow Cells

**Figure 4:**
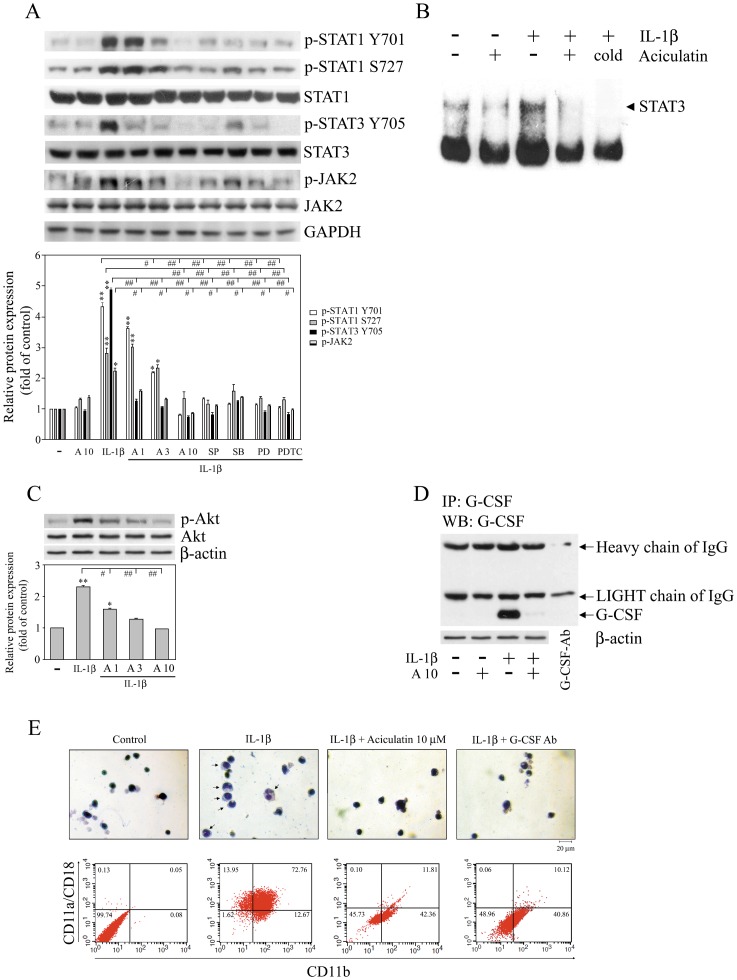
Aciculatin inhibits the phosphorylation of JAK, STAT, and Akt in FLS and the differentiation of 32Dcl3 cells. (A, C, and D) FLS were incubated with 0, 1, 3, or 10 µM aciculatin (A1, A3, and A10) for 30 min, and then for 24 h with 10 ng/mL of IL-1β in the continued presence of aciculatin. Whole cell extracts were then prepared for western blot analysis for the indicated proteins (A and C); equal amounts of cell culture media (“conditioned medium”) were collected and concentrated 10-fold (v/v) (lanes 1–4) or PBS only (lane 5), and then immunoprecipitated with 1 µg of anti-G-CSF antibody, followed by immunoblot analysis using anti-G-CSF antibody or anti-β-actin antibody (as an internal control) (D). (B) FLS were incubated with 0 or 10 µM aciculatin for 30 min, and then for 1 h with 10 ng/mL of IL-1β in the continued presence of aciculatin. The DNA binding activity of the nuclear extracts was then examined in an electrophoretic mobility shift assay using a specific STAT3 DNA probe. (E) Ten-fold concentrated conditioned medium was prepared from FLS incubated with or without aciculatin, and then with IL-1β as in (D), or with IL-1β plus an anti-G-CSF antibody. 32Dcl3 cells were incubated for 10 days with a medium containing 50% of these different conditioned mediums. The cells were then were subjected to Wright-Giemsa staining to detect neutrophils (top row) or washed twice with PBS, incubated at 4°C for 45 min with anti-CD11b FITC-conjugated and anti-CD11a/CD18 PE-conjugated antibodies, and their fluorescence was analyzed by FACScan flow cytometry (bottom row). Magnification  = ×100; scale bar  = 20 µm. In (A) and (C), the extents of indicated proteins expression were quantitated using a densitometer with the Image-Pro plus software, and the relative levels were calculated as the ratios of proteins to GAPDH or β-actin protein levels. The results are expressed as the mean ± SEM, with n = 3. *p<0.05 and **p<0.01 compared with the control group; #*p*<0.05 and ##*p*<0.01 for the comparisons of the groups indicated.

Previous studies have shown that G-CSF binds to the G-CSFR, which leads to activation of the JAK-STAT signaling pathway, and promotes the maturation and differentiation of neutrophils from progenitor cells [28], [29]. We therefore examined whether aciculatin could suppress JAK-STAT signaling and the differentiation of neutrophils from mouse bone marrow cells. As shown in Figure 4A, the treatment of FLS with IL-1β for 24 h caused a significant increase in the phosphorylation of STAT1, STAT3, and JAK2, and these effects were inhibited by aciculatin or by MAPK or NF-κB inhibitors in a concentration-dependent manner. Again, these results suggest that the NF-κB and MAPK pathways play key roles in the inhibition of IL-1β-induced G-CSF activation of the JAK-STAT pathway by aciculatin. Furthermore, a marked increase in STAT3 DNA binding activity was seen in the EMSA assay after the treatment of FLS with IL-1β for 1 h (Figure 4B), and this effect was significantly inhibited by aciculatin (Figure 4B). A recent study showed that G-CSF can also activate the PI3K-Akt pathway in neutrophils and enhance neutrophil motility [30]. As shown in Figure 4C, aciculatin also inhibited IL-1β-mediated (10 ng/mL, 24 h) Akt phosphorylation in a concentration-dependent manner. We next evaluated whether aciculatin had an inhibitory role in the differentiation of neutrophils from progenitor cells. We first determined the levels of G-CSF released by FLS in response to treatment with IL-1β, alone or together with aciculatin, by collecting the conditioned medium and assaying it for G-CSF by using an immunoprecipitation assay. As shown in Figure 4D, significant levels of G-CSF were released by FLS stimulated by IL-1β, and this effect was markedly inhibited by aciculatin treatment. These conditioned media were then concentrated 10-fold, and their effect on neutrophil differentiation tested by incubating mouse bone marrow 32Dcl3 cells with 50% concentrated conditioned medium for 10 days. As shown in the top panels in Figure 4E, the conditioned medium from IL-1β-treated FLS resulted in the appearance of Wright-Giemsa-stained cells with a dense, horseshoe- or ring-shaped nucleus (neutrophils), as shown in a previous study [31], and this effect was not seen using conditioned medium from FLS treated with IL-1β plus aciculatin or with IL-1β plus a G-CSF-neutralizing antibody. In addition, G-CSF has been shown to induce differentiation of precursor cells into neutrophils, which express adhesion molecules, e.g. CD11b/CD18 (Mac-1) and CD11a/CD18 (LFA-1), which are essential for functions of mature neutrophils [19]. As shown in Figure 4E, the incubation of bone marrow cells with conditioned medium from IL-1β-treated FLS for 10 days resulted in a significant increase in Mac-1 and LFA-1 expression, whereas the IL-1β and aciculatin-containing conditioned medium resulted in much lower neutrophil differentiation and Mac-1 and LFA-1 expression by the bone marrow cells, as did conditioned medium containing IL-1β and G-CSF neutralization antibody. Our results clearly demonstrate that aciculatin inhibits myeloid differentiation into neutrophils by reducing G-CSF levels.

## Discussion

Previous studies have shown that IL-1β can stimulate synovial cells to secrete G-CSF [24], [25] and that this induction is regulated at the transcriptional and translational levels, since it can be inhibited by either actinomycin D or cycloheximide [24]. However, the signaling pathway involved is still unclear. In the present study, the IL-1β-mediated increase in G-CSF expression and secretion by FLS was inhibited by aciculatin. Moreover, aciculatin inhibited the IL*-*1β-induced activation of the NF-κB and MAPK pathways. Both of these effects were mimicked by NF-κB/MAPK inhibitors. These results show that the NF-κB and MAPK signaling pathways are involved in G-CSF induction by IL-1β and suggest that aciculatin inhibits the IL-1β-induced G-CSF increase by inhibiting the activation of these pathways. Furthermore, IL-1β treatment of FLS caused an increase not only in G-CSF levels, but also in IL-6, IL-8, and TNF-α levels, and it is known that both NF-κB and MAPK signaling pathways play a role in IL-1β-induced IL-6, IL-8, and TNF-α expression [4], [32]. Our results also showed a differential suppressive effect of aciculatin: it had a greater inhibitory effect on the increase in G-CSF than that of the other cytokines. Moreover, a previous study found that TNF-α treatment of synovial cells also increases G-CSF production, although it is less effective than IL-1β [25]. This raises the possibility that the G-CSF increase was possibly not all as a result of direct IL-1β stimulation, but part may have been due to IL-1β-mediated TNF-α induction. However, in our study, we found that the concentration of TNF-α released by FLS on IL-1β stimulation (51.43±0.82 pg/mL) was much lower than that of 10 nM (170 ng/mL) used to stimulate G-CSF release in this previous study [25]; furthermore, aciculatin treatment also inhibited IL-1β-stimulated TNF-α production. These results therefore indicate that the inhibition of IL-1β-induced G-CSF production by aciculatin occurs primarily by inhibiting the direct effect of IL-1β.

Tissue infiltration by activated neutrophils has been found in inflammatory arthritis [12], [13]. Mature neutrophils play critical roles in antimicrobial defense and innate immunity and contribute to autoimmunity and chronic inflammation, by synthesizing cytokines and chemokines to amplify inflammation [31], [33], releasing B cell activating factor to promote the proliferation and maturation of B cells [34], producing S100 small calcium-binding proteins, such as calgranulin C, to induce proinflammatory effects [35], regulating inflammatory cytokine networks through the action of neutrophil serine proteases, such as cathepsin G, neutrophil elastase, and protease 3 [36], participating in complement production [37] and antibody trafficking [38], etc. G-CSF increases the number of neutrophils by stimulating the expansion of precursors [12], [13], [20], promotes the rate of maturation and release of neutrophils into the circulation [39], induces adhesion molecule expression [17]–[19], and delays the apoptosis of neutrophils [40]. Activated neutrophils expressing Mac-1 and LFA-1 are reported to adhere to the intercellular adhesion molecule (ICAM)-1 and traffic into inflamed joints where they contribute to cartilage damage [18], [41]. G-CSF administration exacerbates collagen-induced arthritis in mice [20] and disease activity is ameliorated by the administration of a G-CSF neutralizing antibody [17]. Further evidence for a pro-inflammatory effect of endogenous G-CSF has been provided by the finding that G-CSF^−/−^ mice are resistant to the induction of acute and chronic inflammatory arthritis [17]. These results demonstrate that endogenous G-CSF is a critical mediator of inflammatory arthritis [17] and suggest G-CSF to be a therapeutic target in inflammatory arthritis [12], [13], [42]. In the present study, IL-1β-induced G-CSF caused bone marrow cells to differentiate into neutrophils and this effect was reduced by G-CSF neutralizing antibody, while, in another study, cell supernatants from IL-1β-treated FLS increased the proliferation of murine myeloid leukemia NFS-60 cells and this increase was blocked by anti-G-CSF antibody [24].

The binding of G-CSF to the G-CSFR triggers the activation of intracellular signaling pathways, including the JAK-STAT [15] and PI3K [16] pathways, and activates PI3K, which enhances neutrophil motility [30]. Moreover, JAK1 and JAK2 are phosphorylated in response to G-CSF induction and activate two DNA binding complexes, a major complex that contains tyrosine phosphorylated STAT3 protein and a minor complex that appears to be the STAT1/STAT3 heterodimer [43]. Another study demonstrated that STAT3 plays a crucial role in granulopoiesis and mature neutrophil function, since STAT3-deficient mice have an aberrant response to G-CSF, showing no increase in circulating neutrophils and an impaired chemotactic response to CXCR2 ligands [28]. In the present study, aciculatin significantly inhibited the G-CSF-triggered phosphorylation of Akt, JAK2, STAT1, and STAT3, supporting the inhibitory effect of aciculatin on neutrophil maturation. Moreover, we also noted that IL-1β treatment of FLS resulted in an increase in IL-6 levels in the culture supernatant, and since IL-6 can activate the JAK-STAT signaling pathway [43], this suggested that the JAK2, STAT1, and STAT3 phosphorylation seen in response to IL-1β stimulation may not be entirely attributable to G-CSF induction. However, aciculatin significantly inhibited IL-1β-mediated G-CSF production, but only slightly inhibited the increase in IL-6 levels. Moreover, the G-CSF neutralizing antibody mimicked the inhibitory effect of aciculatin on neutrophil maturation. These results therefore suggest that the inhibitory effect of aciculatin on the IL-1β-induced activation of JAK2, STAT1, and STAT3, and neutrophil maturation, is primarily due to decreased IL-1β-induced G-CSF expression. Taken together, these findings suggest that aciculatin may have therapeutic potential in the treatment of inflammatory arthritis.

## Materials and Methods

### Materials

Recombinant human IL-1β was purchased from PeproTech Inc. (Rocky Hill, NJ, USA). Rabbit monoclonal antibodies against human IKKα, JNK2, p38, phospho-STAT1 (Tyr701), phospho-STAT1 (S727), STAT1, phospho-STAT3 (Tyr705), STAT3, phospho-JAK2, JAK2, and β-actin were purchased from Epitomics Inc. (Burlingame, CA, USA). Rabbit polyclonal antibodies against human phospho-IKKα (Ser180)/IKKβ (Ser181), phospho-ERK1/2 (Thr202/Tyr204), phospho-p38 (Thr180/Tyr182), phospho-Akt (Ser473), and ERK1/2 and rabbit monoclonal antibodies against human phospho-p65 (Ser536) and phospho-JNK (Thr183/Tyr185) were purchased from Cell Signaling Technology (Danvers, MA, USA). Mouse monoclonal antibody against GAPDH and rabbit polyclonal anti-human Akt were purchased from Santa Cruz Biotechnology Inc. (Santa Cruz, CA, USA). Rabbit polyclonal antibody against p65 was purchased from BioVision, Inc. (Milpitas, CA, USA). Phycoerythrin-labeled anti-mouse CD11a/CD18 antibodies and fluorescein isothiocyanate (FITC)-labeled anti-mouse CD11b antibodies were purchased from BioLegend Inc. (San Diego, CA, USA). Horseradish peroxidase (HRP)- or FITC-conjugated goat anti-mouse, anti-rabbit or anti-rat IgG antibodies were obtained from Jackson ImmunoResearch Inc. (West Grove, PA, USA). Anti-G-CSF antibody was obtained from BioLegend (San Diego, CA, USA). ELISA kits for human G-CSF were purchased from Invitrogen Corp. (Camarillo, CA, USA). The pGL4.32[*luc2P/*NF-κB-RE*/*Hygro] and p5xATF6-GL3 vectors were obtained from Promega Corp. (Madison, WI, USA) and Addgene Inc. (Cambridge, MA, USA), respectively. TurboFect™ *in vitro* transfection reagent was purchased from Fermentas (Burlington, Ontario, Canada). NF-κB, AP-1, and STAT3 EMSA kits were purchased from Affymetrix Inc. (Fremont, CA, USA). All other chemicals were purchased from Sigma-Aldrich (St. Louis, MO, USA).

### Extraction and Isolation

The extraction and isolation method has been described previously [23]. Briefly, whole specimens of the herb *Chrysopogon aciculatus* were collected in Taipei, Taiwan in 2006, and authenticated by Professor Ching-Hsiang Hsieh of the Department of Plant Industry, National Pingtung University of Science and Technology (NPUST). A voucher specimen (no. 70652) was deposited at the Provincial Pingtung Institute Herbarium of NPUST. Whole specimens of *C. aciculatus* were heated under reflux with 95% EtOH for 1 h. After filtration, the EtOH solution was concentrated *in vacuo* to obtain the EtOH extract. The EtOH extract was then partitioned with H_2_O/EtOAc (1∶1) to separate the EtOAc and H_2_O layers. Then, the EtOAc layer was concentrated to obtain the EtOAc extract. It was then chromatographed on a silica gel column, eluted with EtOAc, and further separated on a Sephadex LH-20 column, from which it was finally eluted with MeOH to produce aciculatin.

### Cell Culture

Human FLS were obtained from the synovial tissues of OA patients undergoing total joint replacement surgery after approval by the Ethics Committee of the National Taiwan University Hospital (IRB number: 201106100RC), and the patients gave their written informed consent. Mouse myelomonocytic leukemia WEHI-3 cells and mouse bone marrow 32Dcl3 cells were purchased from the Bioresource Collection and Research Center (Hsinchu, Taiwan). Human FLS and WEHI-3 cells were cultured, respectively, in high glucose Dulbecco’s modified Eagle medium (DMEM) or in Iscove’s modified Dulbecco’s medium (IMDM), supplemented with 10% fetal bovine serum (FBS), while 32Dcl3 cells were maintained in RPMI-1640 medium (all reagents from Invitrogen™ Life Technologies, Carlsbad, CA, USA) supplemented with 10% FBS and 10% WEHI-3 conditioned medium as a source of IL-3. Cells were cultured at 37°C in a humidified atmosphere of 5% CO_2_ in air.

### Cell Viability Assay

Cells (1×10^4^) were incubated with vehicle or aciculatin for 24 h in 100 µl of medium, then 20 µl of a 5 mg/ml solution of 3-(4,5-dimethylthiazol-2-yl)-2,5-diphenyl tetrazolium bromide (MTT) was added and the mixture incubated at 37°C for 2 h. The cells were then pelleted and lysed in 100 µl of dimethyl sulfoxide and the absorbance at 550 nm measured.

### Milliplex® Cytokine Assay

To quantify cytokine production by FLS, culture supernatants were analyzed with a Milliplex® multi-analyte panel kit (Millipore Corp., St. Charles, MO, USA), which allows the simultaneous quantification of the 7 human cytokines IL-1β, G-CSF, IL-6, IL-8, monocyte chemotactic protein-1 (MCP-1), tumor necrosis factor-α (TNF-α), and vascular endothelial growth factor (VEGF). The data were analyzed by using Bio-Plex Manger 4.1.1 software (Bio-Rad, Hercules, CA, USA).

### RNA Isolation and RT-PCR Analysis

Total RNA was isolated from cells using TRIzol reagent (Invitrogen) and single-strand cDNA for a PCR template was synthesized from 5 µg of total RNA using random primers and Moloney murine leukemia virus reverse transcriptase (Promega). The oligonucleotide primers used for the amplification of human G-CSF (GenBank Accession No. BC053585) were sense (2314–2336) 5′-AAC AGC TCA GAG ACC TGT GGC CT-3′ and antisense (2552–2569) 5′-CCA AGG GGC TGG CCT GGA-3′, with a product of 255 bp. The primers for the internal control, β-actin, were sense (613–632) 5′-GAC TAC CTC ATG AAG ATC CT-3′ and antisense (1103–1122) 5′-CCA CAT CTG CTG GAA GGT GG-3′, with a product of 510 bp. Equal amounts (1 µg) of each reverse-transcription product were PCR-amplified using *Taq* polymerase (Promega) and 35 cycles of 1 min at 95°C, 1 min at 58°C, and 1 min at 72°C and the amplified cDNA run on 1% agarose gels and visualized under UV light following staining with SYBR Safe DNA gel stain (Invitrogen).

### ELISA Assay

FLS (1×10^6^ cells) were incubated with or without various concentrations of aciculatin for 30 min prior to stimulation with 10 ng/ml of IL-1β for 24 h in the continued presence of aciculatin, then the culture supernatants were collected and assayed for G-CSF using commercial ELISA kits (Invitrogen Corp., Camarillo, CA, USA).

### Immunoblot Analysis

Cells (1×10^6^) were incubated for 10 min at 4°C in lysis buffer (20 mM HEPES, pH 7.4, 2 mM EGTA, 50 mM β-glycerophosphate, 0.1% Triton X-100, 10% glycerol, 1 mM DTT, 1 µg/ml of leupeptin, 5 µg/ml of aprotinin, 1 mM phenylmethylsulfonyl fluoride, and 1 mM sodium orthovanadate), then were scraped off, incubated on ice for a further 10 min, and centrifuged at 17,000 g for 30 min at 4°C. The supernatants (60 µg of protein) were subjected to SDS-PAGE and blotted onto nitrocellulose membranes, which were then blocked by incubation for 1 h at room temperature with 5% BSA in phosphate-buffered saline (PBS). They were then incubated overnight at 4°C with primary antibody diluted in PBS, then, after three washes with PBS containing 0.1% Tween 20, were incubated for 1 h at room temperature with the corresponding HRP-conjugated antibodies diluted in PBS and bound antibodies detected using an ECL detection kit and exposure to photographic film. Quantitative data were obtained using a densitometer with Image-Pro Plus image analysis software systems (Eastman Kodak Co.).

### Immunoprecipitation Assay

Cell culture supernatants were collected and concentrated 10-fold (v/v) on an Amicon Ultra centrifugal filter device (Millipore, Billerica, MA, USA), then the concentrated supernatant containing 5 mg of protein was immunoprecipitated overnight at 4°C with 1 µg of rat monoclonal anti-G-CSF antibody and A/G-agarose beads (Santa Cruz). The precipitated beads were washed three times with 1 ml of ice-cold cell lysis buffer and 80% of the bound immune complex resolved by 10% SDS-PAGE, followed by immunoblotting using anti-G-CSF antibody, while the remaining 20% the was electrophoresed as above and blotted with anti-β-actin antibodies as the internal control.

### Transfection and Reporter Gene Assay

Cells (5×10^4^) in 1 ml of growth medium were seeded in each well of 24-well plates one day before transfection. Following the manufacturer’s protocol, 1 µg of plasmid pGL4.32[*luc2P/*NF-κB-RE*/*Hygro] or p5xATF6-GL3, which contains the c-fos promoter, and 1 µl of TurboFect™ transfection reagent were mixed for 20 min at room temperature, then were added to the cells and the mixtures incubated for 24 h at 37°C in a humidified atmosphere of 5% CO_2_ in air. Transfection efficiency, determined by fluorescence microscopy, was >60% in all experiments. For the reporter gene assay, 100 µl of reporter lysis buffer (Promega) was added to each well and the cells scraped off the dishes, centrifuged at 17,000 g for 30 s at 4°C, and the supernatants collected. Aliquots of cell lysates (20 µl) were placed in the wells of an opaque black 96-well plate and 40 µL of luciferase substrate (Promega) added and the luminescence immediately measured in a microplate luminometer (Beckman Coulter, Krefeld, Germany).

### Electrophoretic Mobility Shift Assay (EMSA)

Nuclear extracts were prepared and analyzed for NF-κB, AP-1, and STAT3 DNA binding activity using EMSA Gel Shift Kits (Affymetrix) as described previously [23]. In controls, non-labeled probe was used to block the binding of the biotinylated probe. All procedures were performed according to the manufacturer’s instructions.

### Granulocyte Differentiation Assay

FLS (1×10^6^ cells) were treated with 10 ng/ml IL-1β in the presence or absence of 10 µM aciculatin or 2 µg/ml of G-CSF neutralizing antibody for 24 h, then the cell culture supernatants were collected and concentrated 10-fold (v/v) on an Amicon Ultra centrifugal filter device (Millipore, Billerica, MA, USA). For the differentiation assay, 32Dcl3 cells (5×10^3^) were seeded in each well of 24-well plates containing RPMI supplemented with 50% of the above concentrated FLS supernatants and incubated for 10 days, the medium being replaced every day. The progression of granulocytic differentiation was monitored by Wright-Giemsa staining using a kit from Sigma according to the manufacturer’s instructions.

### Flow Cytometry

Expression of CD11b and CD11a/CD18 on differentiated neutrophils was measured by a double-staining flow cytometry assay. After stimulation with 10 ng/ml IL-1β in the presence or absence of 10 µM aciculatin for 7 days, the cells were washed twice with PBS, then incubated for 45 min at 4°C with a mixture of FITC-conjugated anti-CD11b and PE-conjugated anti-CD11a/CD18 antibodies, then, after three washes with PBS, the fluorescent cells were analyzed on a FACScan flow cytometer (Becton Dickinson, Mountain View, CA, USA) using BD CellQuest Pro software (Becton Dickinson).

### Data Analysis

The data were expressed as the mean ± standard error of the mean (SEM) and were analyzed statistically using one-way ANOVA. When ANOVA showed significant differences between groups, Tukey post hoc test was used to determine the specific pairs of groups showing statistically significant differences. A *p* value of less than 0.05 was considered statistically significant. The data were analyzed by using SAS 9.1.3 software.
